# Burnout in Embryologists from the Perspective of an International Expert Panel: A Qualitative Study

**DOI:** 10.3390/bs16060861

**Published:** 2026-05-27

**Authors:** Raquel Urteaga, Amelia Díaz

**Affiliations:** Faculty of Psychology, University of Valencia, Avd. Blasco Ibañez, 21, 46010 Valencia, Spain; raurgar@alumni.uv.es

**Keywords:** embryologists, burnout, stressors, preventive tools, panel of experts, qualitative study, international context

## Abstract

Healthcare professionals, including those working in assisted reproduction, are characterized by high levels of stress and burnout, with embryologists showing the highest levels. The aim of this study was to identify both the stressors that contribute to burnout and the potential prevention strategies they considered useful. For this, a qualitative study based on a semi-structured interview was conducted in a panel of 12 senior embryologists from eight countries in four continents. The interviews were recorded and thematic analysis was performed using MAXQDA^®^ 26 software From a deductive approach, stressors were those inherent to the embryologist profession such as excessive demands; physical and organizational stressors, such as high workload and ergonomic issues; and patient-related stressors, such as difficult communication and interactions. On the other hand, preventive/mitigating factors were classified, according to their nature, as physical (natural light), relational (conflict management), organizational (organizational planning) and psychological (stress management). From an inductive approach, lack of professional recognition arose as an additional theme, where embryologists complained about the unfair situation when comparing themselves with other team members, as healthcare professionals, or even in the wider social context. The results of this work should be considered in interventions that seek to improve the well-being of these professionals by reducing their burnout.

## 1. Introduction

There is a growing global decline in birth rates in developed countries, most of them falling below the replacement level of 2.1 children per woman. The economic, environmental and geopolitical consequences of this declining birth rate make it crucial to examine all factors that may be contributing to or maintaining this situation (Boodoo & Hosein, 2024). One of the contributing factors is undoubtedly infertility associated with increasing maternal age, a consequence of the new social models that prioritize other life goals before family formation (Aitken, 2024). In a worldwide context, infertility, defined as “the failure to achieve a pregnancy after 12 months or more of regular unprotected sexual intercourse”, occurs in one in every six people of reproductive age (World Health Organization, 2023). For this reason, infertility and its management—including the healthcare teams involved in its treatment—must be thoroughly analyzed (Balen et al., 2025).

Healthcare professions are characterized by stressful situations dealing with ill people and their families, both accompanied by high levels of stress. Additionally, healthcare professionals work long hours, including late evenings, weekends and festive days, being exposed to severe diseases that make them more prone to absenteeism because of sickness, mental health issues, and burnout (Dixit & Ghosh, 2019; Williams & Lewis, 2020). The emotional well-being of healthcare teams specialized in assisted reproduction has gained particular attention in recent years due to the increasing demand for these techniques in the treatment of infertility (Boivin et al., 2012; Astro et al., 2025; Cohen et al., 2023; Urteaga & Díaz, 2024). Concern about healthcare professionals’ well-being is reflected in studies such as Fedele et al. (2020), which aimed to understand the subjective perspective of fertility professionals and identify their emotional dynamics within the work environment. Vélez-Muentes and Torres-Jerves (2025) conducted a study whose findings indicate that 70% of staff experience occupational stress, and 60% report not having received workshops or resources to support their mental well-being. Moreover, 80% perceive a negative workplace climate when confronted with challenging situations related to pregnancy failures. Additionally, 70% of employees report an imbalance between work and personal life, and 80% indicate that the center lacks effective strategies to address and manage stress.

Historically, the German American psychiatrist Herbert J. Freudenberger provided the first conceptualization of burnout in 1974, describing it as a psychological state consisting of fatigue and frustration because of unmet professional expectations, commonly observed among care service workers. Professionals experience progressive loss of energy, tiredness, and exhaustion, accompanied by negative cynic and rigid attitudes, and work-related demotivation (Freudenberger, 1974). In 1982, the psychologist Christina Maslach further refined the concept, describing burnout as a gradual process characterized by three work-related dimensions: Emotional Exhaustion, defined as feelings of fatigue, depletion, and lack of emotional energy to cope with daily tasks; Depersonalization, described as a negative, cynic, detached, or indifferent attitude toward work or service users; and Reduced Personal Accomplishment, referring to a perceived loss of professional efficacy and a tendency to negatively appraise one’s performance and abilities (Maslach & Leiter, 1976). Finally, the World Health Organization (World Health Organization, 2019a) defined burnout as “a syndrome conceptualized as resulting from chronic workplace stress that has not been successfully managed.” Burnout is included in the 11th Revision of the International Classification of Diseases (ICD-11) as an occupational phenomenon and is not classified as a medical condition (ICD code Z73.0) (World Health Organization, 2019b).

Previous studies have shown that healthcare teams working on Assisted Reproduction Techniques (ART) experience varying degrees of burnout depending on the country and professional group analyzed (Govardhan et al., 2012; Delaroche et al., 2025; Rausch & Braverman, 2000; Sax & Lawson, 2022). In France, Delaroche et al. (2025) highlighted that over a third of French ART professionals experienced job strain, a condition linked to increased risks of stress, burnout, and health concerns. Among ART healthcare professionals, nurses (Rausch & Braverman, 2000), gynecologists (Govardhan et al., 2012) and psychologists (Sax & Lawson, 2022) documented high levels of burnout. Reported burnout levels in embryologists working in ART have progressively increased over time, in parallel to the increased use of ART worldwide (Balen et al., 2025). In studies performed in Spain, the percentage of embryologists reporting emotional exhaustion, depersonalization and lack of personal accomplishment went from 20%, 15% and 10% respectively in 2014 (López-Lería et al., 2014) to 35.4%, 42.5% and 37.8% ten years later (Urteaga & Díaz, 2024). On the other hand, a comparative study between embryologists working in the United Kingdom (UK) and the United States of America (USA) found higher levels in emotional exhaustion and depersonalization (59% and 62% in emotional exhaustion and 43% and 60% in depersonalization, respectively) whereas the lack of personal accomplishment was lower (15% and 28% respectively) (Murphy et al., 2024).

Although burnout in the ART field has been studied in different professionals and countries, most of the published studies have not associated burnout with specific stressors. Among the exceptions, Basar (2023) conducted an in-depth analysis of factors influencing embryologists working in laboratory settings, highlighting the importance of addressing mental health and work–life balance in reproductive medicine, which may contribute to improving In Vitro Fertilization (IVF) success rates. Urteaga and Díaz (2024), analyzed the level of burnout among assisted reproduction healthcare teams and examined its triggering factors in a quantitative study with a Spanish sample. Results showed that embryologists reported the highest burnout levels as a subgroup within assisted reproduction professionals, and identified three main predictors: workload, communication with patients, and lack of professional recognition. In a more recent work, the same authors (Urteaga & Díaz, 2026) showed a close relationship between burnout and the stressors of workplace pressure and years working in ART.

In the same context, the qualitative study of Astro et al. (2025), about workplace stressors in clinical embryologists in Italy and Spain, found seven sources of stress in the laboratory-based work of embryologists: (1) high workload and a salary that did not match the high responsibility and training required in the job; (2) conflictive relationships with other members of the team, mainly with gynecologists, due to ineffective organization or management in the workplace; (3) highly competitive nature of the profession, resulting from the constant advancement in the techniques, combined with isolation, since laboratory work is performed in small teams or as a single embryologist; (4) lack of professional recognition due to the fact that some of the tasks could be performed by gynecologist or lab technicians, combined with an underdeveloped legislation that does not clarify responsibilities and limits; (5) low patient feedback and short time spent with the patients, making it more difficult to get more comprehensive information about them and develop passion for the profession based on the interaction with patients; (6) making difficult decisions affecting ethical issues when patients are older than 45–50 years and the consequent physical risks in these patients, highlighting the absence and great need of a deontological code for this profession; (7) poor laboratory infrastructure where ergonomic chairs, natural light, adequate bench space and support for the equipment used and dedicated break rooms to rest from the long shifts are highly demanded.

Finally, the study of Basar and Duzcu (2025) based on a general and wide view of the embryologist’s work goes beyond the workplace stressors, presenting the causes of burnout in a classification of two categories: causes of workload-related burnout and causes of mental burnout. Among the stressors related to the workload-related burnout were the excessive workload and time pressures associated with the ART field (where “optimal processing windows” guide the embryologists work, requiring rotatory shifts including early mornings, late evenings, weekends, and holidays, giving place to an unpredictable schedule with periods of very high activity followed by periods of calm or relative low activity, according to seasonal variations in treatment cycles and coordination with physicians and patients), as well as understaffing and resource constraints that are usual in the embryologist workplace. Regarding mental burnout, the more usual stressors or causes proposed were inadequate leadership and low organizational support, poor communication and interdepartmental friction, toxic workplace personalities and team dynamics, limited career advancement and professional development, and emotional labor in reproductive medicine, characterized by feelings of responsibility, guilt and distress as an emotional weight, being aware that the hope of patients to have a family depends on their work.

If the studies of burnout stressors in embryologists are few, those based on strategies or tools to prevent or mitigate burnout are even scarcer, usually as a small part in the discussion of the stressor’s studies (Astro et al., 2025). An exception is again the study of Basar and Duzcu (2025) that analyzed both stressors and causes of burnout as well as strategies to mitigate burnout. Among the tools proposed to mitigate burnout were appropriate staffing; adequate workload distribution; strategic rotation, including recovering periods; work variation, including preference in the tasks but maintaining fairness; and leadership development and training to promote team effectiveness, accompanied by burnout prevention and well-being, mental health resources, and psychological safety, including the incorporation of mental health professionals in the team to implement resilience programs, psychological support and professional assistance in critical situations. At the individual level the interventions proposed included resilience training, stress management, continuous professional and structured career development, effective boundary management between professional and personal life, and adequate workspace design and ergonomics.

Based on these findings, there is a clear need to deepen our understanding of the work situations of these professionals and to expand the scope of research beyond the national studies toward a global study, integrating stressors, preventive/mitigating strategies and any other issue that could affect these professionals’ wellbeing. Although some stressors seem to be common in many studies (Astro et al., 2025; Basar, 2023; Basar & Duzcu, 2025; Urteaga & Díaz, 2024, 2026), others seem more specific to some world zones (Gerrits et al., 2024). The aim of the present study is to analyze the full range of stressors that may be causing or maintaining burnout in embryologists and find out the protective and preventive factors that may help to prevent their onset or mitigate its effects when it is already present. For this, an international expert panel was selected, and semi-structured interviews were conducted. The expert panel was selected based on geographical representation, proven experience as embryologists and having published in the field. Finally, the use of an online semi-structure interview, apart from being a convenient procedure to obtain information from professionals working in different zones of the world, has the advantages of accessing the unique personal perspective of the interviewee rather than a generalized understanding of a phenomenon. At the same time, it is a flexible assessment instrument that provides in-depth information from the interviewees, whilst maintaining a certain structure that allows comparability without preventing the emergence of new themes or aspects (Adeoye-Olatunde & Olenik, 2021).

More specifically, the research questions of this study are the following:

1. What are the stressors that an expert panel of embryologists believe are affecting them?
2. What are the preventive or mitigating strategies they consider the most useful for them?
3. Is there any other theme related to burnout that affects them?

## 2. Materials and Methods

### 2.1. Design

Following the guidelines of Stenius et al. (2008), this study adheres to the three essential criteria for qualitative research. First, the significance of the data was ensured through careful attention to the social and cultural context of the participants (Boodoo & Hosein, 2024; World Health Organization, 2023). Second, data sufficiency and the scope of analysis were addressed by collecting comprehensive information and conducting a thorough examination of emergent themes (Scheepers et al., 2017; Boivin et al., 2012; Gameiro et al., 2012; World Health Organization, 2003). Third, transparency and replicability were maintained through detailed documentation of the research process. To support replication, the full interview protocol is provided as Appendix A.

The choice of a qualitative design was driven by the need to deepen the quantitative analysis of stressors and protective factors associated with burnout in embryologists (Basar, 2023; Delaroche et al., 2025; Urteaga & Díaz, 2024). This design aims to serve as an exploratory study that, based on its findings, allows the development of a survey capable of accurately measuring the constructs of interest. The resulting survey could then be distributed to embryologists worldwide, providing a more precise understanding of the current situation in assisted reproduction laboratories.

### 2.2. Participants

A panel of 14 senior embryologists was generated, covering five continents. Professionals from diverse geographic regions were selected to ensure adequate global representation. Experts were recruited in the context of several Scientific International Conferences on the field of Assisted Reproduction (AR). The consideration of experts was based on their professional positions and a minimum of experience of six years working in AR. All had publications on their field or had been awarded tittles as “Best embryologist” internationally. A total of 14 experts were asked to participate, all agreeing to participate and to record the interview. However, at the time of the analysis and writing of the manuscript, only 12 interviews were conducted; two embryologists were ultimately unable to participate due to personal circumstances that made it impossible to complete the interview within the study’s proposed timeframe.

The expert panel was composed of embryologists working in The United States of America, Spain, the United Kingdom, India, Egypt, Italy, Mexico and Argentina, 6 of them working in private centers and the other 6 in public ones. Most of them were heads or directors of embryology sections in Assisted Reproduction Centers; additionally, 2 were working in universities and 2 in hospitals, and 2 were presidents of AR or Embryologists’ International Associations.

In the recruitment of the experts, the inclusion criteria were to have at least 6 years’ experience as an embryologist and cover the maximum representation of the world continents, finally representing five geographic regions: North America, Latin America, Europe, Asia, and Africa. Each expert in the panel participated independently without consulting other experts in the panel (Kellerhuis et al., 2025).

### 2.3. Materials and Procedure

Following Kallio et al.’s (2016) guide for a semi-structured interview in the frame of a qualitative design, five steps were performed:

(1) Identifying the prerequisites for the use of semi-structured interviews: in this study the semi-structured interview is adequate to capture embryologists’ perceptions and opinions, focusing on issues meaningful for them (burnout) allowing different perceptions due to the participants working in countries from four continents.
(2) Retrieving and using previous knowledge: this phase is based on two elements; firstly, a comprehensive literature review centered on stressors and preventive tools against burnout, and secondly the information obtained by the first author when working as a psychologist in two Assisted Reproduction Clinics in Spain and Italy, recording both potential stressors perceived as contributing to or maintaining professional distress and preventive factors that might improve embryologists’ working conditions.
(3) Formulating the preliminary semi-structured interview guide: the questions were formulated attending the research topics of stressors and preventive tools of burnout in a flexible and open-ended design to guarantee that participants could express their opinion and insight.
(4) Pilot testing of the interview guide: three embryologists known by the first author answered the pilot interview suggesting changes in the order or wording of some questions.
(5) The final interview with 15 questions is presented. Appendix A includes the English version of the interview.

Once the semi-structured interview had been finalized, an initial email outlining the study’s objectives was sent to the selected experts in July 2025, inviting them to confirm their willingness to participate. Upon acceptance, participants received informed consent forms addressing confidentiality and authorization for audio and video recording, as interviews were conducted via the Microsoft Teams platform.

When the informed consent forms had been signed, individual interviews were scheduled with each expert. All interviews were conducted by the first author between July and September 2025. They were recorded, and subsequently transcribed verbatim (Suarez et al., 2013). The first author is fluent in Spanish, English and Italian; therefore, interviews were conducted in Spanish for Spanish speakers, Italian for Italian speakers and English for the remaining participants, all of them fluent in English. The interviews lasted between 20 and 30 min.

The analysis of participants’ responses aimed to identify key stressors and preventive factors, which would serve as the foundation for the development of a survey instrument. This survey constitutes the next phase of the research: a quantitative study to be administered to the largest possible population of practicing embryologists worldwide, a step that will partially contribute to ensuring methodological triangulation in the next stage of the research project, currently in the execution phase.

### 2.4. Ethics

The study was conducted in accordance with the Declaration of Helsinki and approved by the Institutional Ethics Committee of University of Valencia (2024-PSILOG-3468621, 22 October 2024).

### 2.5. Data Analysis

Once the interviews had been recorded, preparation for the qualitative analysis began using the qualitative data analysis software MAXQDA^®^ (VERBI Software, 2026).

First, the interviews were transcribed, focusing exclusively on the question-and-answer segments. Closing remarks and non-essential conversational elements were removed to facilitate systematic handling of the relevant data.

Next, responses to each question were coded with the aim of identifying recurring patterns and shared themes across experts. This coding process enabled the identification of common stressors and preventive factors, which will subsequently be incorporated into the survey instrument to be developed in the next phase of the study.

In the thematic analysis, both authors listened and separately classified information into the two pre-set codes, stressors and preventive/mitigating strategies related to burnout. When a discrepancy happened, each of the authors indicated her point of view and discussed it, many times coming back to the specific expert answer to elucidate the meaning, until an agreement was achieved. The necessity of a third and new code arose from the listening, both authors agreeing on this point.

Therefore, the initial methodological approach was deductive from the codes: stressors associated with burnout and strategies to prevent or mitigate burnout. Thematic analysis was performed on the interviews‘ responses, obtaining data until the saturation point, when no new information was obtained (Saunders et al., 2018). Both authors agreed that this point was reached at interview 11. Interview 12 did not add any new information. However, the study used an inductive approach, allowing the emergence of new themes associated with burnout. Accordingly, the methodological approach implemented in the study was hybrid (Francis et al., 2010).

## 3. Results

The average number of years of professional experience among the expert panel was 17.33 years. Regarding gender, seven (58.3%) were men and five (41.7%) were women. Of the 12 participants, 25% reported having taken medical leave at some point in their careers due to burnout.

The thematic analysis performed used stressors and preventive and mitigating strategies associated with burnout as the initial pre-set codes. From there, the answers of the experts could be classified attending to the stressor context (inherent to the profession, organizational and relational, and patient-related). Similarly, the preventive and mitigating strategies suggested by the experts, those that they considered useful to prevent or fight burnout, could be classified according to their nature (physical, relational, organizational and psychological). This deductive approach was complemented by an inductive one, where the theme of professional recognition arose, affecting both previous themes and being described as a deeper one affecting all aspects of the interviews.

The description of the three codes will be accompanied by some interviewees’ comments and answers (in italics and quotations marks) for a better understanding.

### 3.1. Stress-Related Factors

Table 1 shows the emerging themes for each group of stressors that we explored with the panel of experts.

#### 3.1.1. Qualitative Analysis of the Code: What Inherent Factors in Your Profession Generate the Highest Level of Stress?

The analysis reveals the following key themes:

1. Responsibility and Potential for Errors: A significant source of stress stems from the high level of responsibility associated with the profession, particularly concerning the handling of patients’ embryos and gametes, “*being responsible for the patients’ embryos and their gametes*”. The fear of making errors that could impact a patient’s chance of having a child is a recurring concern. This is highlighted by the response “*there is no room for error or failure*”. The interviewees express anxiety about potential failures in fertilization and the difficulty of explaining these failures to patients. The burden of responsibility is further highlighted by the awareness that a mistake, such as dropping a dish containing an embryo, could have significant consequences. “*Error management*” is explicitly mentioned as a stressor.
2. Workload and Work Pace: The demanding workload and fast pace of work contribute significantly to stress levels. This includes not only the volume of work but also the “*work pace*”, which can be erratic and poorly distributed. The lack of organization and the need to manage multiple tasks simultaneously exacerbate the problem. Embryologists often handle paperwork, work with liquid nitrogen, and perform procedures concurrently, leading to increased pressure and potential for errors. Interruptions, such as phone calls and requests for documents during procedures, further disrupt workflow and increase stress: “*a phone call in the middle of a procedure. Somebody needs a particular document, somebody needs a report*”.
3. Team Management and Interpersonal Dynamics: Managing a team is another source of stress. This includes the “*responsibility of leading a team*” and ensuring a positive “*work environment*”. Understaffing and the need to train junior staff add stress to the workload of senior professionals.
4. Emotional and Physical Demands: The profession takes a toll on both the emotional and physical well-being of embryologists. The long hours, artificial lighting, and awkward postures contribute to physical strain. The emotional burden of dealing with patients’ hopes and dreams, as well as the potential for negative outcomes, can be overwhelming, “*and even though you know you’ve done everything correctly, you’re always left wondering: was it me?*”. The inability to separate personal thoughts and feelings from work can also affect performance. The lack of time for personal life and social interactions further exacerbates stress levels. The interviewees emphasize that “*we’re still human*” and that this aspect is often overlooked.
5. Competition and Private Sector Pressures: In the private sector, competition adds another layer of stress; the “*private sector can be very competitive*”. The pressure to achieve results and the competitive nature of the industry can create a high-pressure environment.

In summary, the inherent stressors in this profession are multifaceted, encompassing high levels of responsibility, demanding workloads, emotional and physical strain, team management challenges, and competitive pressures. These factors contribute to a high-stress environment for professionals in this field.

#### 3.1.2. Qualitative Analysis of the Code: Physical and Organizational Stressors in the Lab

The interviews reveal several recurring themes:

1. Lighting: Inadequate lighting appears to be a significant stressor. One participant explicitly mentions “*the complete lack of sunlight*”. Conversely, a participant notes that the lighting issue was resolved upon moving to a new lab: “*The lighting in the old workspace. Yes, since I changed lab, this problem hasn’t occurred anymore because we work with better artificial lighting*”. Another participant mentions the need to go outside (“*I need two sunbaths a day because you go outside and your eyes seem to appreciate it*”), suggesting a need to compensate for the lack of natural light within the lab. This suggests that both insufficient and potentially harsh artificial lighting can contribute to stress and discomfort.
2. Noise: Noise is another recurring concern. One participant mentions “*It’s also tiring, although it’s true that equipment is becoming quieter*”, indicating that noise from equipment can be a source of fatigue. Similarly, another participant refers to “*equipment noise, for example the noise of hood*”. While one participant seems to have adapted to the noise (“*The noise from the booths, I think that’s gotten to the point that I can’t hear it*”, “*noise yes, but you get used to it*”), the initial impact is still acknowledged as stressful.
3. Ergonomics and Physical Strain: The physical demands of lab work, particularly concerning ergonomics, are highlighted as stressors. Participants mention “*You sit in a particular position, and you cannot move, so it all affects your cervical vertebrae. It affects your lower back*”; “*microscopes where you have to bend your neck*”; and “*back pain from being in a non-natural position for so long*”. The term “*ergonomics*” is directly mentioned by one participant, underscoring the importance of proper workstation design to mitigate physical strain. Prolonged microscope use is specifically identified as a source of stress.
4. Temperature: Temperature control within the lab environment is also noted as a potential stressor, with participants mentioning “*temperature*” and “*temperature management*”. This suggests that maintaining a comfortable and consistent temperature is important for well-being.
5. Isolation and Distractions: The theme of isolation emerges, with one participant mentioning “*Isolation, too. You spend a lot of time alone with your thoughts*”. Conversely, another participant mentions that “*the worst bit about the environment was the distractions*”, and that it was “*really difficult to focus*”. These contrasting viewpoints suggest that the lab environment can be both isolating and distracting, depending on the specific circumstances and individual experiences.
6. Workload and Pressure: The comments also touch upon the impact of workload and high-pressure environments. One participant mentions “*the disorganized and great workload*”, while another refers to “*working under incredibly, essentially high-pressure environment*”. These factors contribute to the overall stress experienced in the lab.
7. Prolonged Working Hours: Prolonged working hours, often under demanding conditions, are identified as a significant stressor: “*prolonged working hours at the microscope*”. This is compounded by factors such as limited breaks and repetitive tasks, further exacerbating physical and mental strain.

In summary, the analysis reveals that physical and environmental factors in the lab, such as inadequate lighting, noise, poor ergonomics, temperature issues, isolation, distractions, workload, pressure, and prolonged working hours, contribute significantly to the stress experienced by these professionals. Addressing these issues is crucial for promoting a healthier and more productive work environment.

#### 3.1.3. Qualitative Analysis of the Code: Stress Factors Related to Patients

The primary stressors related to patients can be categorized into three main themes:

1. Unrealistic Expectations and Managing Expectations: This is the most frequently mentioned stressor. It involves patients having expectations that are too high or not aligned with the realities of treatment. This includes the expectation of guaranteed success, pregnancy at the first attempt, or an overestimation of what the clinic can achieve. Managing these expectations, especially when the outcome is negative, is a significant challenge. The stress arises from the pressure to meet these unrealistic expectations and the difficulty in communicating the limitations of the treatments. Interviewees feel the need to “lower expectations” and present a realistic view, which can be difficult when patients are not receptive; “*they come to us with essentially unrealistic expectations*”. The optimistic discourse sometimes used in consultations may contribute to these unrealistic expectations.
2. Emotional Burden and Lack of Support for Patients: The emotional impact of patients’ experiences, particularly their sadness and lack of emotional support, is a major stressor. This includes feeling the patients’ pain, dealing with their anxiety and the emotional pressure of the process. Interviewees often take the emotional burden home, feeling responsible for the outcomes and remaining invested even after the procedures; “*for me the hardest thing about patients is their sadness and the lack of support that they get for their emotional needs*”. Giving bad news and managing the emotional fallout is particularly stressful. The lack of support for patients exacerbates these feelings, making it harder for professionals to cope with the emotional intensity.
3. Difficult Patient Interactions and Communication Challenges: This involves challenges in communicating with patients, particularly when delivering bad news or managing difficult behaviors. It also includes dealing with patients who are not receptive, rude, or do not understand the information provided. The stress is compounded by the need to explain complex information and manage the patients’ emotional reactions, especially when they are unprepared for potential negative outcomes. Some interviewees expressed a need for training in how to communicate with patients effectively. “*Bad manners are particularly difficult to tolerate*”.

In summary, the most significant stress factors related to patients are unrealistic expectations, the emotional burden and lack of support, and difficult patient interactions. Addressing these areas could potentially reduce stress and improve the well-being of professionals in this field.

### 3.2. Preventive Factors

Table 2 shows the emerging themes for each group of preventive factors that we explored with the panel of experts:

#### 3.2.1. Qualitative Analysis of the Code: Physical Tools for Burnout Prevention

The interviewees suggest several physical tools and resources that could help prevent or mitigate burnout:

1. Ergonomic Furniture: Several interviewees emphasized the importance of ergonomic furniture, particularly chairs. One person mentions the need for industry to work on more ergonomic furniture, specifically for microscope work: “*Chairs specifically designed for working at the microscopes*”. Another participant highlights the difficulty of finding chairs with adequate ergonomics. The consideration of the staff’s physical posture at the microscope when purchasing furniture is also mentioned as a key factor.
2. Comfortable and Relaxing Spaces: Providing comfortable relaxation areas or break rooms is a recurring theme. This includes creating “*a comfortable relaxation area/break room*” and “*comfortable spaces where people can gather*”. The idea is to offer spaces where employees can take breaks and recover from prolonged microscope use.
3. Natural Light and Ventilation: Access to natural light is considered important. One interviewee suggests trying to improve natural light conditions, while another mentions the benefit of having a place with natural light to sit, especially for those working in basements. The lack of sunlight and ventilation is noted as a negative aspect of the work environment. Ultraviolet filters for natural light are also mentioned
4. Workspace Design and Size: The physical design and size of the workspace are also brought up. One interviewee contrasts cramped laboratories with the ideal of a larger space, while another simply mentions “*the laboratory design*”.
5. Equipment: Some interviewees mention specific equipment that could improve the work environment, such as a radio or television in the break room.

These suggestions reflect a desire for a more comfortable, ergonomic, and pleasant physical work environment. Addressing these physical factors could contribute to reducing burnout among employees and increase their wellbeing.

#### 3.2.2. Qualitative Analysis of the Code: Relational Tools for Prevention

The interviews reveal a strong emphasis on relational tools as a means of prevention, particularly in the context of burnout and stress management among embryologists and related staff, identifying the following key themes:

1. Importance of Open and Honest Communication: Several segments highlight the necessity of “*sincere and clear communication*” within teams. This involves creating a safe space for team members to express their feelings and concerns without fear of reprisals. For instance, one interviewee suggests weekly team sessions “*to see what aspects could be improved about work without reprisals*”. The goal is to foster a culture of honesty where individuals feel comfortable addressing challenges and working collaboratively towards solutions.
2. Team Building and Group Cohesion: Team-building activities are frequently mentioned to strengthen bonds and improve relationships among colleagues. These activities can range from informal gatherings outside of work to more structured group meetings and therapy sessions. The benefits of group cohesion include increased support, improved communication, and a greater sense of belonging. For example, one interviewee mentions implementing “*group meetings once a week*” to foster a stronger sense of team unity. Another interviewee shares that their clinical manager “*brought us all together for group therapy to improve the internal relationship between colleagues*”. Also, having breakfast all together is mentioned as a relational tool.
3. Training in Communication and Conflict Management: Several interviewees emphasize the need for training programs focused on communication skills and conflict management. This type of training can equip staff with the tools they need to navigate difficult conversations, resolve conflicts constructively, and communicate effectively with both colleagues and patients. One interviewee suggests “*more training courses for people in every department, depending on what specific aspects they face. So, conflict management at the end of the day*”. Another interviewee highlights the importance of training embryologists to communicate with patients, noting that “*embryologists who are coming up should have training to actually communicate like they should; they should talk to patients and staff members*”.
4. Appreciation and Recognition: Showing appreciation for the work of embryologists and other staff is identified as an important relational tool. This can involve recognizing their contributions, acknowledging their expertise, and providing opportunities for professional development. One interviewee stresses the need for “*an awareness of the head of the team, just say, hey, everyone here is important*”.
5. Empathy and Understanding: Several segments underscore the importance of empathy and understanding in fostering positive relationships. This involves being attentive to the needs and concerns of colleagues, and creating a supportive environment where individuals feel valued and respected. One interviewee suggests that “*you cannot expect or force other people to think like you, but you can expect them to at least understand your needs*”.

In conclusion, the interviews suggest that relational tools play a critical role in promoting well-being and preventing burnout among embryologists and related staff. By fostering open communication, encouraging team building, providing relevant training, and cultivating a culture of appreciation and empathy, clinics can create a more supportive and resilient work environment.

#### 3.2.3. Qualitative Analysis of the Code: Organizational Tools for Prevention

The interviews reveal a strong emphasis on organizational tools and strategies, highlighting the following key themes:

1. The Need for Improved Organization and Planning: Several interviewees highlight the necessity for better organization and planning to manage workload and reduce stress. This includes optimizing work schedules to avoid overburdening staff on certain days and ensuring adequate staffing levels to handle the workload. The sentiment is echoed by the need for someone in operations to oversee planning and improve processes. ‘Organization’ is mentioned as something to improve.
2. Importance of Communication and Coordination: Effective communication and coordination among team members and different departments are crucial for preventing inefficiencies and improving patient care. The lack of communication can lead to frustration for both staff and patients, as highlighted by the example of patients having to contact multiple departments for a single answer.
3. Staff Recognition and Support: Recognition of staff efforts and providing adequate support are essential for preventing burnout and maintaining morale. This includes not only material appreciation, such as salary and resources, but also moral appreciation, as an interviewee said, “*just proper recognition, I think that’s important*”. Additionally, ensuring access to well-trained professionals and sharing the workload can alleviate the burden on individual team members.
4. Management Responsiveness and Feedback: The need for management to be responsive to staff feedback is a recurring theme. Creating a safe environment where employees can voice their concerns (“*we need our managers to listen to us without making us feel bad about it*”) without fear of negative repercussions is crucial for identifying and addressing organizational issues.
5. Resource Availability and Allocation: Adequate resources, including materials and equipment (“*enough materials and equipment, are a necessity*”), are necessary for efficient operation and preventing shortages that can lead to stress and burnout. This includes ensuring a sufficient stock of disposable items and media, as well as having enough incubators and microscopes.
6. Work–Life Balance and Time Off: Respecting employees’ time off and ensuring they can take vacations is crucial for their well-being. The ability to take breaks during the year and proper scheduling of vacation time contributes to reducing stress and preventing burnout, “*especially the organization of vacation days*.”
7. Resistance to Change: The interviews also reveal challenges associated with implementing organizational changes: “*there are many things we are still doing in a very outdated way, and they need to be changed, but people find change difficult*”. Resistance to change can hinder efforts to improve processes and adopt more efficient ways of working.

In summary, the analysis indicates that effective organizational tools for prevention encompass a range of strategies, including improved planning and scheduling, enhanced communication and coordination, staff recognition and support, responsive management, adequate resource allocation, and respect for work–life balance. Addressing resistance to change is also crucial for successful implementation of these tools.

#### 3.2.4. Qualitative Analysis of the Code: Psychological Tools for Prevention

The psychological themes were less explicit than the organizational ones, but the following can be inferred from the concerns raised by the interviewees:

1. Recognition and Appreciation: The need for “*proper recognition*” and “*moral appreciation*” directly addresses the psychological well-being of employees. Feeling valued and acknowledged for their work can significantly reduce burnout and increase job satisfaction. This goes beyond mere financial compensation and includes acknowledging the importance and difficulty of their work.
2. Reducing Stress and Burnout: Several segments implicitly address psychological well-being by focusing on factors that contribute to stress and burnout, such as “*access to counselling or therapy sometimes*”. These include heavy workloads, lack of support, and feeling overwhelmed. Addressing these issues through organizational changes can have a positive impact on employees’ mental health. One interviewee proposed “*the presence of an on-site psychologist*”.
3. Open Communication and Feedback: Creating a safe space for employees to voice their concerns and providing feedback is crucial for addressing psychological stressors: “*you need to have proper communication with your team*”. When employees feel heard and understood, they are more likely to feel supported and less likely to experience burnout.
4. Work–Life Balance and Respect for Time Off: Respecting employees’ holidays and ensuring they have opportunities for breaks and time off is essential to prevent burnout and promote psychological well-being. This allows employees to recover and maintain a healthy balance between work and personal life.

In summary, while not explicitly stated, the text segments suggest that psychological tools for prevention include recognizing and appreciating employees, reducing stress and burnout through organizational changes, promoting open communication and feedback, supporting work–life balance, and addressing resistance to change. These elements contribute to a healthier and more supportive work environment, ultimately promoting the psychological well-being of employees.

### 3.3. Professional Recognition

When the inductive approach was introduced in the analysis, an exploratory third code arose related to the perceived level of lack of professional recognition experienced by embryologists compared with other professionals in their respective work environments. The segments coded with this theme reveal a complex and often contradictory landscape, where recognition varies significantly based on geographic location, institutional context, and even individual career trajectory. Overall, the segments suggest that professional recognition is often insufficient, particularly when considering the importance of the embryologist’s role in the outcome of assisted reproductive technologies (ART).

Here is a breakdown of the key aspects of this theme:

#### 3.3.1. Lack of Professional Recognition

1. General lack of recognition: Many interviewees express a sense of insufficient professional recognition. An embryologist from Spain said “*There is no legal recognition, no salary recognition, no social recognition, and no recognition even within the very structure of a fertility clinic*”. Another from Mexico added, “*If you go to Mexico, for example, the embryologist simply does not exist*”. This lack of recognition manifests in various forms: legal recognition, inadequate salaries, and a general undervaluing of the profession within the clinical setting.
2. Lack of Recognition as Healthcare Professionals: Several segments highlight the fact that embryologists are not always recognized as healthcare professionals, which has implications for their roles and responsibilities. In Spain, for example, embryologists are not considered healthcare personnel (“*if we feel recognized? not at all, we’re not even healthcare personnel*”), yet they are tasked with responsibilities such as reviewing serologies and genetic counseling, which are typically reserved for those with healthcare credentials.
3. Disparity in Recognition Compared to Clinicians: A recurring sentiment is that embryologists work “behind the scenes” and that most of the recognition goes to the clinicians: “*there’s all these cards and chocolates from patients thanking their doctor and their nurse*”. Patients often express gratitude to doctors and nurses but rarely acknowledge the embryologist’s crucial role in complex procedures like Intracytoplasmic Sperm Injection (ICSI).
4. Lack of Social Recognition: There is a lack of social recognition, with many people unaware of the existence and importance of embryologists: “*No recognition, because people still don’t understand what we do*”; “*there’s never a single mention of the person that sat there and did the ICSI for three hours*”.

#### 3.3.2. Geographic Variations in Recognition

1. Variations Across Countries: The level of recognition varies significantly from country to country. While some European countries, like Italy, have officially recognized the profession of embryology (“*it is well recognized, also because there was an official communication from our Ministry in 2018 that acknowledges the profession of embryologist as a healthcare profession. We are still one of the few countries in Europe where it is formally recognized*”), others, like Mexico, do not even acknowledge the existence of embryologists (“*The embryologist is highly undervalued in Mexico*”), whereas Argentina is presented as a more favorable environment where embryologists are respected and sought after, but even there, there is room for improvement. In Egypt, embryologists are well regarded and often mistaken for doctors. In USA the embryologist’s professional recognition seems to be higher.

#### 3.3.3. Factors Influencing Recognition

1. Advocacy and Awareness Efforts: Some interviewees are actively working to change the lack of recognition: “*I’ve done quite a lot of work in the social media space in the UK, think the work with the charity is really important*”. These efforts aim to raise awareness about the embryologist’s role and contributions to ART.
2. Professional Advancement: Achieving a leadership position can significantly impact individual recognition: “*I am right now going to become the chair of The Embryology Society. So obviously things are going to be very different*”.
3. Clinic Structure: The structure of a clinic can influence recognition, with some clinics possibly undervaluing the embryologist’s role. As an interviewee said, “*there is no recognition even within the very structure of a fertility clinic*”.

#### 3.3.4. Progress and Optimism

1. Recent Progress: Some interviewees note that there has been progress in recent years, with people starting to recognize the importance of dedicated professionals working in the labs: “*It’s only in the recent 4–5 years that people have started to sit back and take notice of the fact that, you know, there are these dedicated professionals that work inside the labs*” or “*definitely there’s progress*”.
2. Efforts to Change the Status Quo: There are active efforts to change the lack of recognition through advocacy and awareness campaigns. “*I think I’m doing my hardest to change that. And I do think it’s working*”.

#### 3.3.5. Impact of Lack of Recognition

1. Potential for Systemic Issues: One interviewee suggests that a strike by embryologists would cause the collapse of ART units (“*the units would sink*”), highlighting the critical role they play and the potential consequences of their lack of recognition.
2. Data Entry Issues: Because embryologists are not always considered healthcare professionals, there can be issues with handling of data and compliance with regulations in the healthcare system, “*because only healthcare personnel are allowed to do it. And interestingly, embryologists are an exception*”.

In summary, the segments coded with “professional recognition in your field is sufficient” reveal a nuanced and often challenging situation for embryologists. While there are instances of recognition and progress, the overall sentiment suggests that professional recognition is often lacking, particularly when compared to other healthcare professionals in the field of ART. This lack of recognition has implications for the profession’s status, compensation, and ability to contribute fully to patient care. The countries’ differences highlight the need for advocacy and standardization to ensure that embryologists receive the recognition they deserve across different world regions and healthcare systems.

**Figure 1:**
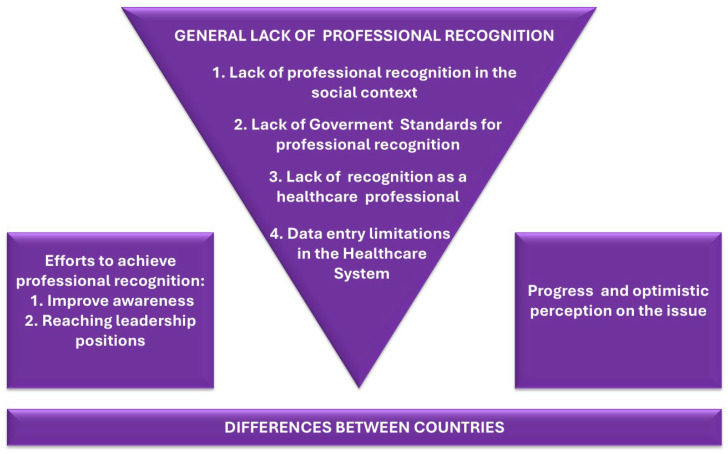
Key aspects of professional recognition.

Figure 1 illustrates the more relevant elements in the theme of lack of recognition. The lack of recognition is presented in an inverted triangle in a descendent direction from a more general context to a more specific one. The lack of general recognition is followed by lack of recognition in the social context, followed within the national context, then by the lack of clear government standards for the profession (which, in many cases, leads to embryologists not being considered healthcare professionals), and finishing with the contradiction that, despite not being listed as healthcare professionals, in many cases they are allowed to handle, as an exception, very confidential patient data within the healthcare system. Most of the elements that made up the figure depend on the existence or not of National Regulations and Standards issued by the corresponding governments and the recognition of embryologists as healthcare professionals. Two additional aspects have been introduced on the issue, on the left and right sides of the inverted triangle. The optimism and perception of progress on the issue is situated on the right, where some countries have finally recognized embryologists as healthcare professionals (Italy would be one of such cases), whilst the continued efforts that embryologists make to achieve professional recognition, through charities, awareness campaigns, trying to achieve leadership position and other initiatives, is represented on the left.

**Figure 2:**
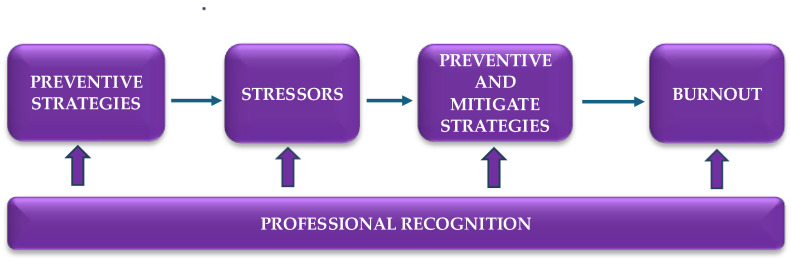
Scheme of stressors, preventive and mitigating strategies, professional recognition and burnout, based on Stress Model (Lazarus & Folkman, 1987).

Figure 2 presents a tentative scheme, based on the Lazarus and Folkman Transactional Stress Model (Lazarus & Folkman, 1987), showing how the three factors in this study could work. Preventive strategies could avoid or limit the effect of stressors affecting embryologists, whereas both preventive and mitigating strategies could mediate and lower the levels of the stressors already present and prevent the effect of future stressors with burnout as the dependent variable. From the analysis performed, lack of professional recognition could work as a stressor that is difficult to classify as specifically organizational, relational, physical or inherent to the profession, as it seems to run at a deeper level and affect all the others, as if lack of professional recognition makes the embryologist’s work not real, as if being “*behind the scenes*” means that “*they do not exist*”. This is why when embryologists are asked about strategies to prevent or mitigate burnout, professional recognition is all-pervading, coming up as a prerequisite to any other preventive or mitigating strategy.

## 4. Discussion

The progressive decline in birth rates, the rising prevalence of infertility, and the increasing reliance on ART over recent decades constitute an undeniable global challenge (Aderaldo et al., 2023; Hanevik & Hessen, 2022; Hariton et al., 2023). This has led to an important increase in assisted reproduction procedures in most countries, resulting in an increase in the workload of healthcare professionals working in this field (Kocourková et al., 2014, 2023). Different studies have shown that professionals working in AR presented high burnout percentages, with embryologists being those with the highest burnout levels among them (Murphy et al., 2024; Urteaga & Díaz, 2024).

The objective of this study was twofold: first, to identify what the stressors are that a panel of senior embryologists reported as more associated with burnout, and second, to find out what strategies and tools to prevent or mitigate burnout the expert panel deemed necessary. Additionally, a third element arose in the results, the lack of recognition of embryologists as a profession in two main contexts, as healthcare professionals and in the wider social context.

Many stressors identified in this qualitative analysis have been reported previously in several studies (Astro et al., 2025; Basar, 2023; Basar & Duzcu, 2025; Boivin et al., 2017; Delaroche et al., 2025; Urteaga & Díaz, 2024), with some of them inherent to the embryologist profession, such as high responsibility, workload and work pace or conflict in team management; some related to patients, such as managing patients’ expectations, emotional burden or problems in the interaction and communication with patients; and some more related to physical and organizational aspects, including lighting, noise, temperature, space, distractions, isolation or lack of ergonomics in the equipment, but also organizational aspects such as workplace pressure and prolonged working hours.

What makes this study more complete, in a similar way to that of Basar and Duzcu (2025), is that apart from stressors, it included questions about preventive factors or strategies that embryologists consider useful in reducing burnout, preventing its appearance or mitigating its effects when it is present. Among these physical preventive tools, ergonomic furniture, natural light and the availability of spaces to relax and recover are included; in the relational and organizational tools, communication training, team cohesion and good organization and planning are highlighted; finally, among the psychological ones, the management and reduction of stress and burnout, the recognition and appreciation of their work and open communication are the main tools identified by the interviewees. Some of these preventive strategies match those previously reported by Basar and Duzcu (2025).

The physical/environmental and organizational stressors identified in this study match and overlap those proposed in recent studies (Astro et al., 2025; Basar & Duzcu, 2025; Urteaga & Díaz, 2024, 2026). For example, in the physical and environmental stressors, we found that embryologists referred to specific elements, such as the ergonomics of chairs and the problems with noise (very often generated by laboratory equipment), that could lead to interruptions and distraction and higher probability of errors, alongside the importance of natural light for professionals working in a lab most of the time, the necessity of time in well-appointed break rooms to recover from intensive work at the microscope or the isolation that this type of work could produce in them. The organizational stressors, some of them included in the stressors inherent to the profession in the analysis, such excessive responsibility, workload, pressure, conflicting relations with other team members and prolonged working hours, are also frequently found in other recent studies. One of the themes found is the patient-related stressors, an aspect that seems to concern embryologists for several reasons: the patients’ expectations, the communication with them when the news is not good, the emotional burden and lack of support, and difficult patients are aspects that represent an extra emotional weight for embryologist that Basar and Duzcu (2025) highlighted in their work.

Regarding prevention, psychological support and training to prevent and mitigate the effects of stress and burnout is a preventive strategy found in this study and proposed by Basar and Duzcu (2025). Embryologists’ mental health and workplace wellbeing are very relevant aspects to consider when proposing tools or interventions for embryologists. When the preventive strategies are analyzed, there is a close correspondence with the stressors, in the sense that the control of stressors through preventive strategies could reduce the burnout in these professionals. For example, the implementation of physical factors such as ergonomic chairs, natural light, noise and temperature control, and enough space in the lab exactly corresponds with the specific stressors reported. Similarly, having more embryologists working in the clinics, allowing rotations and avoiding prolonged working hours and working on weekends or holidays, is a strategy that specifically addresses the overload and work pace stressors. Training programs in stress management, in communication or in mediation could have a favorable effect on the professionals’ stress perception and the response to it, on patient–professional communication and leveling patients ‘expectations, and on reducing conflicting situations between the team members. However, although there are many preventive tools that could address organizational, physical, relational stressors, or stressors inherent to the embryologist profession, when burnout is present, as many studies have shown (López-Lería et al., 2014; Murphy et al., 2024; Urteaga & Díaz, 2024, 2026), apart from identifying and controlling the specific stressors affecting the professionals, psychological or medical care should be available in order to treat and mitigate burnout symptomatology (Tavella et al., 2021) or other symptoms that usually accompany it, such as medical conditions, depression and anxiety (Kakiashvili et al., 2013; Laurent, 2017).

Recognition is a recurrent theme in relational and psychological preventive tools, and in fact, our analysis identifies professional recognition as an exploratory third theme, which indicates the importance that this aspect has for embryologists. It is clearly a dimension that warrants further in-depth examination, as limited specific evidence has been found on this topic. Nevertheless, the results suggest that insufficient professional recognition contributes to increased burnout in these professionals. The recognition theory (Honneth, 1995; Renger et al., 2017) put forward three forms of social recognition. Achievement-based esteem, where the achievement and contribution made by a person is socially recognized, would be the first one. The second one would be equality-based respect in which the recognition would be based on the respect of each person’s equal basic rights and dignity and, finally, need-based care, understood as a fulfillment and care for each person’s emotional needs, would be the third one. Renger et al. (2020) found that each type of recognition was closely related to the three burnout dimensions: emotional exhaustion, depersonalization and lack of personal accomplishment. In other words, social esteem in the workplace seems to increase personal accomplishment; respect, care and fulfillment of emotional needs could decrease depersonalization; and, finally, equal basic rights and dignity would be associated with decreased emotional exhaustion. Apart from the close relationship of lack of professional recognition with the three dimensions of burnout, where it seems to play a broad role, lack of professional recognition has been a general complaint in all the interviews, reported as a stressor associated with burnout, and as a preventive/mitigating strategy to reduce or prevent burnout. Therefore, the professional recognition of embryologists should be carefully addressed in prevention strategies aimed at reducing burnout and preventing disengagement or even, potentially, the abandonment of the profession.

The lack of recognition of embryologists as a healthcare profession and the absence of specific regulations regarding roles and responsibilities in their profession seem to be a source of stress, especially when compared to clinicians and other healthcare professionals working in ART. Lack of recognition is a common complaint in countries where there are no standard regulations, such as Mexico or Spain among others, where the embryologists’ work is not shown, where they are always kept hidden behind the work of other clinicians in the AR contexts, or where their work is undervalued, with lower salaries than other health professionals and working in conditions that seriously interfere with family or social life as consequence of the particular requirements of their work (Astro et al., 2025; Basar, 2023). A tentative model has been proposed based on Lazarus and Folkman’s stress model adapted to the dimensions included in this study. Professional recognition seems to be closely related to all dimensions; it is a recurrent complaint and is perceived as a stressor associated with burnout both in countries where there is no formal recognition and even in those where there is formal recognition, but where the embryologist work is undervalued and poorly recognized. Additionally, professional recognition appears as a key element in the proposal of strategies to prevent or mitigate burnout, made by the expert panel as it influences relational, organizational and psychological strategies. Accordingly, it represents a key element to be considered in any burnout intervention, independently from the context from which it arises.

The strengths of this work can be divided into three parts. Firstly, the aim of the study is not only to identify stressors related to burnout but also the tools and strategies that senior embryologists propose to prevent or reduce burnout. The second strength is the inclusion criteria used, trying to cover a wide variety of countries from five world regions, Europe, North America, South America, Asia and Africa. Finally, and not least important, the methodology used in the study, a qualitative study with a semi-structured interview following strict steps to ensure replicability, is another strength.

Nevertheless, this study presents also some limitations; the number of senior embryologists that made up the panel could have been bigger, representing more global zones such as Australia. Additionally, we should consider that many countries have not been assessed corresponding to wide zones. For example, there are very few studies focusing on Africa, where Gerrits et al.’s (2024) study focusing on the sub-Saharan Africa region is one of the exceptions. This study examined the practices of embryologists in assisted reproductive clinics across sub-Saharan Africa, highlighting the challenges faced in providing fertility care in low- and middle-income countries. The interviews conducted illustrated the multiple tasks they perform on top of their laboratory work: entrepreneurial tasks, advocacy, training, development of regulations, mentoring and patient counseling, including the shortage of embryologists in the region, the need to set up ‘first’ clinics in their respective countries, the lack of trained counselors in clinics and the mobility of IVF staff. Context is important in shaping these practices. Lack of methodological triangulation and limited saturation assessment are also limitations of this study. Only data saturation has been applied in the present study, and methodological triangulation will be partially solved when the worldwide survey in progress has finished. Finally, the analysis was performed separately by both authors, discussing and coming back to the interview answers, until an agreement was achieved, with no external or independent judgment, a procedure that could affect the objectivity of the results. All these limitations suppose a limited generalizability of the results of the study.

The initial information about the most common stressors identified by senior embryologists in our expert panel, together with the preventive strategies they proposed, should be followed by future studies on their implementation. One important aspect our study has highlighted is that of the professional recognition of embryologists working in AR. This has been found to be a major issue for most of the embryologists interviewed, and it is an issue that should be solved in the future. The European Society of Human Reproduction and Embryology (ESHRE) Working Group on Embryologist Training Analysis found that only in 12 countries, out of the 31 European countries that are part of the society, the profession of clinical embryologist is recognized by the government or competent authorities, and only 6 countries had a scientific society specifically representing the clinical embryologist profession, whilst in 13 countries embryologists had no recognition whatsoever (Scarica et al., 2023). This situation should change, not only in Europe but worldwide. Finally, having acquired qualitative information about stressors and preventive tools related to burnout in embryologists, the next step should be to create a survey based on this information to be completed by embryologists from all over the world in a quantitative study, to complete our information about these professionals around the world. This survey should cover the stressors found here, in a structured order considering the three categories: stressors inherent to the profession, relational/organizational stressors and patient-related stressors. In a similar way, the survey should ask for the strategies embryologists consider adequate to prevent/mitigate burnout in the four categories grouped in this study, attending to their nature: physical, relational, organizational and psychological. Finally, attention should be focused on professional recognition, mainly in the social and healthcare context.

## 5. Conclusions

The present study was designed to obtain firsthand information about what embryologists from different parts of the world consider as the stressors affecting their work, and which preventive and mitigating strategies they consider as the most adequate. Stressors found in other studies have also surfaced in the present study, such as high responsibility, work pressure, lack of ergonomic furniture or emotional burden, accompanied by their complementary proposals of preventive and mitigating strategies, such as improving communication, good organization management, reduction in stress and treating burnout. Additionally, a third theme emerged, professional recognition, a dimension that seems to be influencing the remaining dimensions. Lack of professional recognition is perceived as a stressor and, conversely, achieving proper professional recognition could represent an important preventive strategy with a relevant role in the mitigation of burnout.

## Figures and Tables

**Table 1:** Stressors associated with burnout and emerging themes included in each stressor.

Profession-Inherent	Physical and Organizational	Patient-Related
1. Responsibility and potential for errors	1. Lighting	1. Unrealistic expectations and managing expectations
2. Workload and work pace	2. Noise	2. Emotional burden and lack of support for patients
3. Team management and interpersonal dynamics	3. Ergonomics and physical strain	3. Difficult patient interactions and communication challenges
4. Emotional and physical demands	4. Temperature	
5. Competition and private sector pressures	5. Isolation and distractions	
	6. Workload and pressure	
	7. Prolonged working hours	

**Table 2:** Burnout-preventive factors by groups and emergent themes.

Physical Tools	Relational Tools	Organizational Tools	Psychological Tools
1. Ergonomic furniture	1. Importance of open and honest communication	1. The need for improved organization and planning	1. Recognition and appreciation
2. Comfortable and relaxing spaces	2. Team building and group cohesion	2. Importance of communication and coordination	2. Reducing stress and burnout
3. Natural light and ventilation	3. Training in communication and conflict management	3. Staff recognition and support	3. Open communication and feedback
4. Workspace design and size	4. Appreciation and recognition	4. Management responsiveness and feedback	4. Work–life balance and respect for time off
5. Equipment	5. Empathy and understanding	5. Resource availability and allocation	5. Addressing resistance to change
		6. Work–Life balance and time off	
		7. Resistance to change	

## Data Availability

The datasets generated and analyzed during the current study are available from the corresponding author on reasonable request.

## Abbreviations

The following abbreviations are used in this manuscript:

ICD	International Classification of Diseases
ART	Assisted Reproductive Technologies
AR	Assisted Reproduction
USA	United States of America
UK	United Kingdom
ESHRE	European Society of Human Reproduction and Embryology

## Appendix A. Semi-Structured Interview

1. How would you define burnout in one sentence?

2. How many years have you been working in Assisted Reproductive Technology (ART)?

3. How many times have you felt “burned out” or emotionally exhausted at work?

4. Have you ever taken sick leave due to burnout or emotional exhaustion?—If yes, how long did it last?

5. Which physical/environmental aspects of the laboratory generate the most stress for you?

6. Which psychological aspects of the laboratory work do you find most draining?

7. Which elements of your profession cause you the highest stress levels?

8. Do you think your salary is adequate for the work you do?—If not, why?

9. Do you think your professional role is well recognized?—If not, why?

10. What aspects related to patients cause you the most stress?

11. What is your happiest moment during a typical workday in the lab?

12. What is your most difficult or challenging moment in the lab?

13. How would you describe your relationships with the rest of the medical team?

14. What tools or resources do you think are necessary to prevent burnout in your work?—Psychological tools—Relational tools—Organizational tools—Environmental/Physical tools

15. Would you add any questions to this interview? Do you feel like something is missing?
